# Social Innovation for Food Security and Tourism Poverty Alleviation: Some Examples From China

**DOI:** 10.3389/fpsyg.2021.614469

**Published:** 2021-05-04

**Authors:** Guo-Qing Huang, Fu-Sheng Tsai

**Affiliations:** ^1^College of Economics and Management, Southwest University, Chongqing, China; ^2^North China University of Water Resources and Electric Power, Zhengzhou, China; ^3^Department of Business Administration, Cheng Shiu University, Kaohsiung, Taiwan; ^4^Center for Environmental Toxin and Emerging-Contaminant Research, Cheng Shiu University, Kaohsiung, Taiwan; ^5^Super Micro Mass Research and Technology Center, Cheng Shiu University, Kaohsiung, Taiwan

**Keywords:** social innovation, food security, tourism poverty alleviation, China, food safety

## Abstract

The COVID-19 pandemic has brought hunger to millions of people around the world. Social distancing measures coupled with national lockdowns have reduced work opportunities and the overall household incomes. Moreover, the disruption in agricultural production and supply routes is expected to continue into 2021, which may leave millions without access to food. Coincidentally, those who suffer the most are poor people. As such, food security and tourism poverty alleviation are interlinked when discussing social problems and development. While the corporate interest in tourism poverty alleviation is as old as the industrial revolution, little research has been conducted to show how social innovation can be leveraged to reinforce food security and alleviate poverty. Thus, this case study examines the food industry in rural China to establish how it conducts social innovation in food production and distribution to facilitate social development and mitigate poverty.

## Introduction

Rural China was selected for the study because, while poverty in the country has dropped immensely over the past decade, it remains a major problem in the countryside. Historically, China’s countryside has been disproportionately taxed and receives fewer benefits compared to the urban regions despite the numerous economic success and development across China. Although agriculture is the primary occupation for people living in the villages, the produce generated is mostly consumed locally and rarely makes it into the market. The social innovations discussed in this case study encompass the novel practices that focus on meeting a community’s social needs in a better way than the existing solutions. Today, these innovations are necessary for mitigating the looming global food crisis that may arise due to COVID-19. The case study is significant because it shows how the food industry can utilize innovations to reinforce the current food production and distribution systems while facilitating social development and reducing poverty.

Global risk such as the COVID-19 pandemic discussed in this article is associated with social innovation for tourism poverty alleviation significantly. The design and implementation of social innovation should be adhered to the nature of the COVID-19 as an overall context. In the pandemic situation, the most vital imperative is to allow the whole society’s normal functionality in more distant way while not losing the advantages of physical contact for people. Many areas of revolutionary actions should be taken in the society when it deals with social innovation in the global pandemic, such as public governance, education, and personal life improvement. For example, a better remote, electronic government system might facilitate efficiency for citizens to handle their public affairs while lessening the need of physical contact via crowd/cluster gathering. The same can be applied to the distant learning education and distant medical care areas. Therefore, it is necessary to discuss the impact of global emergent risk events that make food insecurity and poverty more serious. This article tends to make a conceptual analysis with practical examples to demonstrate both what have been done and possibilities in promoting tourism poverty alleviation through social innovation in the context of global pandemics.

## Analysis of the Problem

The majority of children who grow up in poverty are more likely to have less educational opportunities, lower literacy levels, as well as suffer from undernourishment. The problem in rural China is worsened by the fact the parents who manage to move into urban areas in an attempt to change their economic conditions are not allowed by the government to take their children with them (Song et al., 2019). As a consequence, the current government policies and practices have largely failed in ensuring food security and mitigating poverty within the countryside (Paudel Khatiwada et al., 2017; Ma et al., 2019). However, the private sector has made significant contributions to China’s food industry through social innovation, specifically in food production and distribution.

Unlike the Western world where frozen food consumption is the norm, the Chinese, especially the older generations and those living in the rural areas, prefer fresh meats, live seafood, and seasonal vegetables and fruits (Nath et al., 2015). However, with the COVID-19 pandemic causing disruptions in food production and distribution, people are forced to change their consumption habits in favor of frozen foods (Zhong et al., 2018). In recent years, the wet markets, where fresh produce is sold, are gradually losing ground to supermarkets in China. While the conventional markets may remain the primary source of fresh food for most Chinese people, social innovation led by supermarkets is helping in changing consumer perceptions on frozen foods. Freezing is one of the effective social innovations within the food industry (Falasconi et al., 2019). However, its success in alleviating poverty depends on how well the people living in rural China embrace it.

The current problem of food insecurity in rural China may deteriorate as the global economy suffers from the 2020 economic depression due to the COVID-19 pandemic. The most vulnerable region with regard to food security is in the western counties that mainly comprise of cluster villages. The authors further note that poor natural conditions coupled with fragile ecologies make it difficult to maximize yields and eliminate the risk of food shortages. Natural conditions are the major issues undermining food security in China. The climatic conditions and geography often restrict agricultural production, particularly in the Western provinces where the majority of people live below the poverty line (Nath et al., 2015; Tianming et al., 2018). Additionally, poor natural conditions coupled with natural disasters weaken the local grain production. As a result, most of these regions experience acute shortages of food and even animal feed (He et al., 2016). The challenging environment and poor living conditions also cause a high incidence of disease (Filiberto and Gaunt, 2013). Moreover, the poor people in these rural regions lack the sufficient knowledge necessary to increase their nutrition and health outcomes. Additionally, topographical conditions create unique challenges in the development and maintenance of transportation facilities. These and other problems have a significant negative impact on the overall food insecurity in rural China, a situation that is expected to continue unless major social innovations are made.

## Social Innovation in Food Productions and Distributions

Social innovation in food production and distribution remains one of the most effective ways of solving the food insecure and poverty problems in rural China. The need to come up with social practices based on food is emerging in response to the numerous uncertainties within the current industrial food system (Haberman et al., 2014). The recent changes within China’s economic and environmental conditions over the past decade have challenged the global security of food supplies. As a result, there have been several and important social innovations concerning food across China, especially within the rural regions. These social innovations include increasing smallholder farmers’ connectivity gap with urban markets, improving digital social innovation and E-agriculture in support of the Belt and Road Initiative (BRI), establishing a community-supported agriculture model, embracing artificial intelligence (AI) and blockchain technology, and achieving social innovation by optimizing waste prevention strategies through the Food Use for Social Innovation by Optimizing Waste Prevention Strategies (FUSIONS) project.

### Increasing Smallholder Farmers’ Connectivity Gap With Urban Markets

One of the problems identified involves the fact that most of the farmers in the ruler areas lack easy access to markets. As a result, the majority of their produce is consumed locally and rarely gets sold in the market, especially in urban areas where the demand is highest. Consequently, the economic status of small-scale farmers remains below the poverty line despite working tirelessly in their farms. To address this issue, the food industry in China conducts social innovation through the United Nations Food and Agriculture Organization (FAO) Innovation Lab and the AgLabCx center. These innovation hubs give young scientists in China an opportunity to brainstorm and come up with solutions aimed at addressing smallholder farmers’ connectivity gap with urban markets (Food and Agriculture Organization of the United Nations, 2019). To address the prevailing issue with regard to distribution and connectivity gap, the FAO and the World Food Programme (WFP) partnered with the AgLabCx center as well as the Tsinghua University toward a socially innovative project named Delivering Together for Sustainable Development Fund (Beckford et al., 2011; Food and Agriculture Organization of the United Nations, 2019). The project mobilized talented young scientists and students to address the connectivity gaps through co-creation workshops. The project was implemented in three phases. Frist, tech-companies, E-commerce practitioners, researchers, as well as smallholder farmers were brought together to brainstorm on how to address connectivity issues relating to trust-building between customers and producers, increasing the capacity for smallholder farmers, and sharing crucial market data.

Second, the main connectivity gaps were identified based on the preliminary analysis conducted. Finally, an 8-week postgraduate service design course was designed and administered through the Innovation Lab. The primary objective was to help Tsinghua students to develop practical solutions that directly addressed the current connectivity gaps. Three recommendations were suggested and approved by the government for countrywide implementation. The first one involved creating an online platform where small-scale farmers could directly market their products to potential urban buyers. Second, a farmer–consumer exchange platform was designed to increase engagement and trust. Finally, a farmer–technical expert instant communication app for small-scale farmers was developed to offer support throughout the production and distribution processes. Thus, the innovation hubs have been effective in giving young innovators in China an opportunity to brainstorm and come up with solutions aimed at addressing smallholder farmers’ connectivity gap with urban markets.

### Digital Social Innovation and E-Agriculture in Support of the Belt and Road Initiative

One of the consequences of increased globalization is that the level of cooperation between countries becomes more important. As a result, the BRI launched in 2013 by China, with its agriculture development component, has the potential to improve food production and distribution locally, regionally, as well as globally (Khan et al., 2018). An initiative is a form of social innovation with far-reaching implications across the country’s poverty levels and food security (Foggin, 2018). For example, the initiative brings market opportunities and development to the remote rural areas in China which have historically been left out from the global expansion in trade for the past four decades (Foggin, 2018). However, for the intuitive to achieve effective rural development and alleviate poverty, the infrastructure investments alone are not sufficient.

While infrastructure investments are necessary, their overall impact within the food industry with regard to food productions and distributions depends on several other factors. First, well-designed measures that benefit both women and men are likely to complement these infrastructure investments, especially in tourism poverty alleviation (Sternberg et al., 2017). As a result, in 2017, the Chinese government began widespread campaigns aimed at encouraging investments from the private sector in activities such as logistics, agricultural production, and storage to complement the BRI. The efforts from the private sector were necessary as they ensured the investments and business models adopted relied on an inclusive and sustainable framework (Foggin, 2018). As such, private agricultural investments not only benefited the poor farmers in rural China but also provided the local communities with food sectary.

As is the case with transportation infrastructure, enhanced communication technology and information sharing capabilities have the potential to mitigate poverty and enhance food security in China. This is demonstrated by the increased efficiencies and connectivity brought by E-agriculture, and invaluable social innovation. E-agriculture innovations have provided small-scale farmers in rural areas with the opportunities of making more money and reducing wastages, since all their produce is sold (Mwalupaso et al., 2019). For example, improved access to information has had a significant impact on small-scale farmers across rural China where poverty levels have dropped by 20% between 2017 and 2019 (Ishangulyyev et al., 2019). E-commerce is probably one of the most powerful applications of ICT in food production and distribution (Tianming et al., 2018). Thus, increasing ICT capabilities is a form of social innovation which can range from establishing knowledge and information systems to increasing agricultural productivity or from accessing financial services to using ICT in farmer organizations. All these ICT capabilities improve risk management and food safety, and even strengthen rural governance. Thus, new developments in digital agriculture may hold the key to addressing local, regional, and global food security challenges (Teo et al., 2019). However, the effectiveness of these technologies relies on how well they are tailored to work for social justice and eliminate the digital divide between rural and urban populations.

In the context of digital social innovation and E-agriculture in support of the BRI, FAO together with the International Telecommunication Union (ITU) and China’s Ministry of Agriculture and Rural Affairs is now working to reinforce the current E-agriculture solutions in the country. For example, the 2018 E-agriculture Solutions Forum held in Nanjing, China, was attended by more than 200 delegates from over 30 countries. The forum was the first of its kind in China and provided a unique opportunity for the country to identify the proven E-agriculture solutions that can benefit agriculture stakeholders in rural China as well as share knowledge on successful solutions that can help in mitigating poverty and food insecurity. Between 2018 and 2020, China has identified and implemented effective E-solutions which have helped rural farmers scale up their production and connect with potential customers from the urban centers (Foggin, 2018). Therefore, the digital social innovation and E-agriculture in support of the BRI have been effective in addressing the food and poverty issues affecting rural China.

### The Community-Supported Agriculture Model

Another way in which China’s food industry conducts social innovation with regard to food productions and distributions is through a community-supported agriculture model. They are defined as an innovative approach in producing sustainable food by bridging the urban–rural gap to effectively connect consumers to farmers (Food and Agriculture Organization of the United Nations, 2019). The models began emerging in the 1960s in Japan following the Minimata disaster, where water pollution by the Chisso chemical manufacturer caused severe mercury poisoning among 2,000 people (Sakamoto et al., 2018). Following the disaster, most of the seafood was contained but soon entered the global food supply chain (Chen and Zhou, 2020). As a result, a network among local housewives in Japan was developed to help members source their food directly from organic farmers (Miyamoto, 2018). Eventually, these networks progressively became global, with Urgenci, an online platform uniting millions of local producers and consumers (Inoue, 2019; Tang et al., 2019; Struś et al., 2020). These networks are examples of a community-supported agriculture model and are effective in facilitating social development and reducing poverty within rural China because consumers commit themselves to buy from local producers regularly.

One of the social innovations advanced by Chinese food producers and consumers involves the promotion of green ecological agriculture which is largely based on the community-supported agriculture models. For instance, the annual community-supported agriculture conference is perceived as the highest policymaking platform in China (Samoggia et al., 2019). The 10th annual conference on community-supported agriculture was held in 2018 in Sichuan province, Zhanqi Village and attracted over 300 scientists and practitioners (Tang et al., 2014; Vassalos et al., 2017; Damayanti et al., 2018; Samoggia et al., 2019). The impact of community-supported agriculture on local markets is mostly positive (Krul and Ho, 2017). Similarly, the small-scale farmers are less successful when farming alone without utilizing community-supported platforms (Savarese et al., 2020). As such, the fact that community-supported agriculture plays an essential role in facilitating social development and reducing poverty within the society is enough motivation for farmers to participate in such models (Chen et al., 2019). Thus, community-supported agriculture models are essential in promoting green ecological agriculture and mitigating food insecurity and poverty.

### Artificial Intelligence and Blockchains

More aggrotech start-ups in China are developing learning models than ever before. AI is one of the most powerful tools in answering China’s challenges in food security and poverty (Freeman et al., 2019). As a social innovating, AI takes into account the limited availability of arable land as well as climate change issues in China (Zeng et al., 2012). For example, the Plantix, a new cloud-based AI application, was launched in China to help farmers detect diseases in their crops (Vermeulen et al., 2018). The app provides an opportunity for furthering social development and reducing poverty as it helps small-scale farmers monitor and deal with diseases effectively. Additionally, the application is simple to use and does not require users to possess complex skills in technology. In most cases, such applications are designed to help illiterate farmers to track the productivity of their farms simply and easily (Vermeulen et al., 2018). In the case of the Plantix app, farmers are required to have a smartphone for taking pictures of their crops and then uploading them into the platform with GPS locations. The application then provides a diagnosis to the farmer showing any pest, disease, or nutrient deficiency. The application leverages AI tools to make a diagnosis automatically. Thus, as more aggrotech start-ups in China continue developing AI learning models, the rate of social innovation in food productions and distributions is expected to grow further, thereby facilitating social development and reducing poverty.

Blockchains are a new paradigm in growing trust and increasing transparency in China’s food industry. Blockchains are a ledger of accounts and transactions which are stored and written by all participants (Hanson et al., 2017). As such, it provides a reliable source of truth about the state of farms, available contracts in farming, and inventories, where the collection of such information is often expensive (Duan et al., 2020). The blockchain technology can track the provenance of food which helps in creating trustworthy food supply chains as well as reinforcing trust among consumers and producers (Giungato et al., 2017). As a trusted social innovation focused on storing data, blockchains facilitate the use of data-driven technologies to make farming smarter and more productive which facilitates social development and reduces poverty. Moreover, blockchains can be jointly used with AI to allow timely payments between stakeholders. The applications of blockchain technology in food supply chains are far-reaching and range from agricultural insurance to smart farming and market transactions (You et al., 2013; Mao et al., 2018; Jin et al., 2020). Thus, blockchains are an effective social innovation within the food industry which helps in mitigating food insecurity and poverty in rural China.

### Social Innovation by Optimizing Waste Prevention Strategies

Finally, the FUSIONS project has played an essential role in strengthening food productions and distribution across rural China. The project was initiated in August 2012 and lasted 4 years up to 2016 (Ishangulyyev et al., 2019). Its overall aim was to contribute to the harmonization of food waste monitoring as well as validate the feasibility of social innovative measures in optimizing food use within the food supply chain (Ishangulyyev et al., 2019). China was among the countries that adopted the recommendations suggested by the project with regard to utilizing social innovations to minimize food waste (Aschemann-Witzel et al., 2015). The following are the major recommendations implemented by the Chinese government to alleviate food insecurity and mitigate poverty. First, government agencies focus on gathering reliable data to develop a criterion for monitoring food waste. The approach provides governments with an opportunity to assess food waste quantities to map any emerging trends that can hinder or reinforce the measures put in place to prevent and reduce food waste (Reynolds et al., 2016). As such, one of the unintended benefits of adopting these recommendations is improved environmental, economic, and social impacts.

The recommendations provided by the FUSIONS project require countries to comprehensively model their existing trends with regard to social innovations in the food supply chain from a knowledge-based perspective such as research and technology trends. Doing so establishes appropriate multi-stakeholder platforms the local and national levels which work together toward mitigating food insecurity and poverty (Borrello et al., 2017). Finally, the project highlights the impotence of the private sector in contributing to policymaking with regard to food security. The most effective socially innovative solutions incorporate perspectives from both the private and the public sectors (Diaz-Ruiz et al., 2018). Consequently, the recommendations made in the project have and continue playing a significant role in identifying new ways of using food surpluses that would otherwise have gone to waste. China has been achieved this through the development of new food products as well as through innovations aimed at redistributing excess foods to charities (Jurgilevich et al., 2016; Wang et al., 2016; Song and Cho, 2017). Overall, China’s vision in implementing the recommendations was focused on securing a more resource-efficient economy with minimal food waste. Thus, the FUSIONS project has played an essential role in helping China develop and implement social innovations aimed at strengthening its food productions and distribution systems across rural areas.

## Conclusion

Food security and poverty are interlinked, thus, social innovations within the food industry must be focused on mitigating both issues rather than forcing on one, especially in a special global situation of pandemics. Social innovation in food production and distribution remains one of the most effective ways of solving food insecurity and poverty problems in rural China. The primary social innovations in China include increasing smallholder farmers’ connectivity gap with urban markets, improving digital social innovation and E-agriculture in support of the BRI, establishing a community-supported agriculture model, embracing AI and blockchain technology, and achieving social innovation by optimizing waste prevention strategies through the FUSIONS project. The need to formulate additional social innovations will help respond to the numerous uncertainties within the current industrial food system. Nonetheless, this article only focused on rural China to offer clear demonstration of the abovementioned issues, which limits the generalizability of the arguments made. Thus, we humbly suggest that future analyses are needed on comparative discussions across different countries in the world that have the same situation as that of urban China.

## Author Contributions

G-QH and F-ST contributed to writing—original manuscript. Both authors contributed to the article and approved the submitted version.

## Conflict of Interest

The authors declare that the research was conducted in the absence of any commercial or financial relationships that could be construed as a potential conflict of interest.
