# Corrigendum: Social Innovation for Food Security and Tourism Poverty Alleviation: Some Examples From China

**DOI:** 10.3389/fpsyg.2021.712709

**Published:** 2021-06-10

**Authors:** Guo-Qing Huang, Fu-Sheng Tsai

**Affiliations:** ^1^College of Economics and Management, Southwest University, Chongqing, China; ^2^North China University of Water Resources and Electric Power, Zhengzhou, China; ^3^Department of Business Administration, Cheng Shiu University, Kaohsiung, Taiwan; ^4^Center for Environmental Toxin and Emerging-Contaminant Research, Cheng Shiu University, Kaohsiung, Taiwan; ^5^Super Micro Mass Research and Technology Center, Cheng Shiu University, Kaohsiung, Taiwan

**Keywords:** social innovation, food security, tourism poverty alleviation, China, food safety

There is an error in the Funding statement. The new statement should be: “Supported by the national social science fund project Research on Path Design and Policy Support for Rural Tourism in Ethnic Areas to Consolidate the Achievements of Poverty Alleviation (20BSH062), the Fundamental Research Funds for the Central Universities (SWU2109207).”

The authors apologize for this error and state that this does not change the scientific conclusions of the article in any way. The original article has been updated.

